# NLRP6 self-assembles into a linear molecular platform following LPS binding and ATP stimulation

**DOI:** 10.1038/s41598-019-57043-0

**Published:** 2020-01-13

**Authors:** Fangwei Leng, Hang Yin, Siying Qin, Kai Zhang, Yukun Guan, Run Fang, Honglei Wang, Guohui Li, Zhengfan Jiang, Fei Sun, Da-Cheng Wang, Can Xie

**Affiliations:** 1 National Laboratory of Macromolecules, Institute of Biophysics, Chinese Academy of Sciences, Beijing, 100101 China; 2 State Key Laboratory of Membrane Biology, Laboratory of Molecular Biophysics, School of Life Sciences, Peking University, Beijing, 100871 China; 3 Department of Biological Chemistry and Molecular Pharmacology, Harvard Medical School, Boston, MA 02115 USA; 4 Department of Medicinal Chemistry and Molecular Pharmacology, Purdue University, West Lafayette, IN 47907 USA; 5 State Key Laboratory of Protein and Plant Gene Research, Peking-Tsinghua Center for Life Sciences, College of Life Sciences, Peking University, Beijing, 100871 China; 6 Laboratory of Molecular Modeling and Design, State Key Lab of Molecular Reaction Dynamics, Dalian Institute of Chemical Physics, Chinese Academy of Sciences, Liaoning, 116023 China; 7 High Magnetic Field Laboratory, Key Laboratory of High Magnetic Field and Ion Beam Physical Biology, Hefei Institutes of Physical Science, Chinese Academy of Sciences, Hefei, 230031 China

**Keywords:** Inflammation, Innate immunity, Electron microscopy

## Abstract

NOD-like receptors (NLRs) localize in the cytosol to recognize intracellular pathogen products and initialize the innate immune response. However, the ligands and ligand specificity of many NLRs remain unclear. One such NLR, NLRP6, plays an important role in maintaining intestinal homeostasis and protecting against various intestinal diseases such as colitis and intestinal tumorigenesis. Here, we show that the major component of the outer membrane of gram-negative bacteria, lipopolysaccharide (LPS), binds NLRP6 directly and induces global conformational change and dimerization. Following stimulation by ATP, the NLRP6 homodimer can further assemble into a linear molecular platform, and ASC is recruited to form higher molecular structures, indicative of a step-by-step activation mechanism. Our study sheds light on the mystery of LPS-induced inflammasome initiation, reveals the architecture and structural basis of potential pre-inflammasome, and suggests a novel molecular assembly pattern for immune receptors.

## Introduction

The innate immune system is essential in the first line of defense against microbial infection, tissue injury and regulating the adaptive immune system. The initiation of innate immune responses relies on the recognition of conserved structures or products of pathogens by pattern recognition receptors (PRRs). Toll-like receptors (TLRs) and newly identified nod-like receptors (NLRs) are key PRRs in the innate immune system. TLRs recognize a wide variety of microbial products outside the cell or in intracellular endosomes and lysosomes and NLRs localize in cytosol to detect intracellular pathogen products^1^. Twenty-two NLRs have been identified in humans and all share typical tripartite domain architecture: the N-terminal domain recruits downstream effector molecules by homotypic protein-protein interactions; the central nucleotide-binding oligomerization (NOD, or NACHT) domain enables activation of the signaling complex via nucleotide-dependent oligomerization; and the C-terminal leucine-rich repeat (LRR) domain recognizes microbial ligands in a manner analogous to TLRs. Studies on NOD1, NOD2, NLRP1, NLRP3, NLRX1 and NLRC4 suggest a direct interaction between NLRs and cytoplasmic pathogen- and damage-associated molecules^2–9^, however, ligands and ligand specificity remain poorly understood for the majority of NLRs.

**Figure 1:**
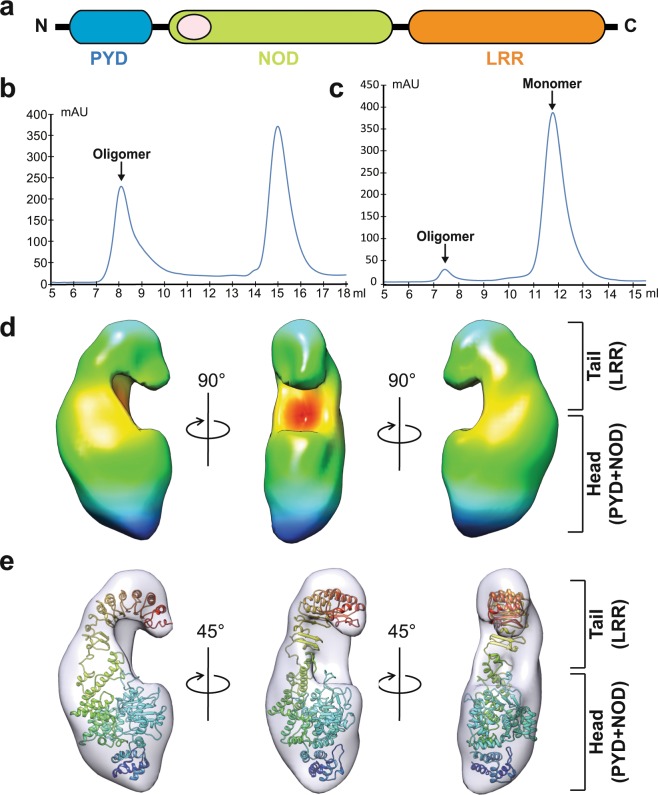
Structure of NLRP6 monomer. (**a**) Schematic of the 2D domain structure of NLRP6. (**b**) Full-length human NLRP6 was purified as putatively activated oligomers. (**c**) Homogenous monomer preparations were obtained from NLRP6 oligomers. (**d**) 3D reconstruction of NLRP6 monomer, shown in three orientations. A characteristic horseshoe-like shape at the top indicates the location of LRR domain. (**e**) A computational model of NLRP6 monomer was built based on the closest homologous structure of NLRC4 (LRR and NOD domain: 4KXF, PDB) and PYD domain of NLRP6 (6NCV, PDB), and modelled into the 3D-EM density map. See also Supplementary Table 1, Supplementary Fig. 1.

Following ligand binding and activation, intracellular NLRs assemble into multimeric molecular platforms such as inflammasomes and trigger innate immune defenses^6,10^. Our knowledge of inflammasomes is mainly derived from studies of NLRP1, NLRP3 and NLRC4, whereby these NLRs form inflammasomes with the adaptor ASC and Caspase-1, promoting the maturation of the inflammatory cytokines IL-1b and IL-18^6,10,11^. NLRP6 (Fig. 1a) is one of 14 pyrin domain-containing members of the NLR family and participates in inflammasome assembly^12–16^. A major focus of the last decade has been detailing the immune response and signaling pathway induced by NLRP6^12,17–19^. Recent years, NLRP6 has been reported to sense viral RNA, and Lipoteichoic Acid from Gram-positive pathogen, and serve as a negative regulator of neutrophil-mediated host defense during Gram-positive bacterial infection in the lungs through regulating both neutrophil influx and function, suggesting a multifaceted role of NLRP6^20–22^. Here, we show that NLRP6 can also recognize LPS. NLRP6 monomer can form homodimer upon LPS binding and further self-assemble into a linear molecular platform by ATP stimulation.

## Results

### Purification and 3D-EM reconstruction of NLRP6 monomer

Recombinant full-length human NLRP6 (1–892) was purified from *Escherichia coli* strain BL21 (DE3) as putatively activated oligomers (Fig. 1b). The final homogenous monomer preparations were obtained from NLRP6 oligomers by switching buffer conditions (Supplementary Methods) and size exclusion chromatography (Fig. 1c), and used for the following experiments. A 3D electron microscope (EM) density map was reconstructed using 16128 particles (Supplementary Table 1), revealing an overall figure ‘6’ structure for the NLRP6 monomer (Fig. 1d and Supplementary Fig. 1) with a horseshoe-like shape at the top indicating the location of the LRR domain. A computational model of the NLRP6 monomer used for map fitting was constructed based on the closest homologous structure for NLRC4 (LRR and NOD domain: 4KXF, PDB) and the PYD domain of NLRP6 (6NCV, PDB). With minor adjustment of the orientation between the LRR domain and NOD domain, the final model is largely consistent with a previously published NLRC4 structure^23^ and fit our 3D electron microscopy map well (Fig. 1e). To our knowledge, this is the first view of the full-length molecular architecture of NLRP6.

### LPS is able to bind NLRP6 directly and induce its dimerization

**Figure 2:**
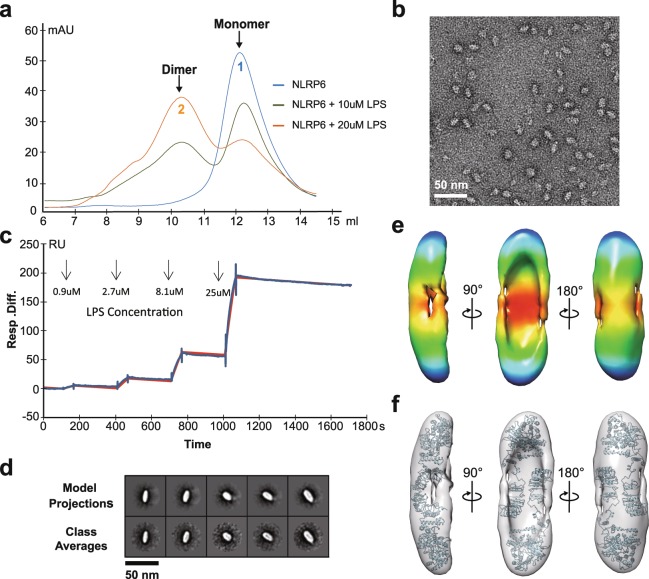
LPS induce the dimerization of NLRP6 monomer. (**a**) Gel filtration analysis of NLRP6 monomer (blue), and NLRP6 monomer incubated with 10uM LPS (green) and 20uM LPS (orange) in Superdex 200 size exclusion chromatography. (**b**) Representative raw image of LPS-induced NLRP6 dimer. Bars = 50 nm. (**c**) The affinity between LPS and NLRP6 was measured by SPR experiment and single cycle kinetics method. Data fits a 1:1 Langmuir binding model well. Blue color is the experimental data, and Red color is the fitted curve. (**d**) Comparison of representative experimentally determined class averages (below) to 2D reprojections of the reconstructed 3D volume (above) of NLRP6 dimer. (**e**) 3D reconstruction of NLRP6 dimer shown in three orientations. (**f**) Model fitting of the NLRP6 dimer. Two LRR domains with horseshoe-like shapes are located around the two caves of the EM density map, interacting with each other in an antiparallel manner. See also Supplementary Table 2, Supplementary Fig. 2.

Binding of agonistic ligands to the LRR sensor domain may lead to activation of NLRs prior to oligomerization^24^. We used analytical gel filtration to identify potential ligands of NLRP6 and characterized direct ligand binding features. A panel of various microbial components (Table 1) was selected to incubate with the NLRP6 monomer (Peak 1, Fig. 2a) and only lipopolysaccharides (LPS Ra, L9641, Sigma, Supplementary Fig. 2B) were found to specifically induced a peak shift in NLRP6 (Peak 2, Fig. 2a). The resulting hydrodynamic radius change suggests dimerization of NLRP6 upon LPS stimulation, confirmed by EM measurement of the same preparation (Fig. 2b). The ratio of dimer formation is positively correlated with the concentration of LPS when incubated, suggesting a dose-dependent interaction between LPS and NLRP6 (Fig. 2a). We further measured affinity using surface plasmon resonance (SPR) with single cycle kinetics^25^ (Fig. 2c) and the equilibrium constant (KD, 7.58 × 10^−8^ M) indicates specific and high-affinity binding between LPS and NLRP6 (Supplementary Table 2).

**Table 1:** Microbial components tested in ligand screening experiments.

Microbialcomponent	Product code/company	Molar ratio^a^
C12-iE-DAP	tlrl-nodkit2, InvivoGen	1:20
E-DAP	tlrl-nodkit2, InvivoGen	1:20
L18-MDP	tlrl-nodkit2, InvivoGen	1:20
MDP	tlrl-nodkit2, InvivoGen	1:20
M-Tri_DAP_	tlrl-nodkit2, InvivoGen	1:20
M-Tri_LYS_	tlrl-nodkit2, InvivoGen	1:20
PGN-Ecndiultra pure	tlrl-nodkit2, InvivoGen	1:20
PGN-Sandi ultra pure	tlrl-nodkit2, InvivoGen	1:20
Tri-DAP	tlrl-nodkit2, InvivoGen	1:20
Murabutide	tlrl-nodkit2, InvivoGen	1:20
LPS (Ra)	L9641, Sigma	1:20
dsDNA	Synthetic^b^	1:20
ssDNA	Synthetic^b^	1:20
Natural DNA	Benzonase, Merck^c^	1:20
Natural RNA	Benzonase, Merck^c^	1:20

^a^Molar of NLRP6/Molar of Microbial component = 1:20.

^b^25-mers dsDNA and 25-mers ssDNA used in ligand screening experiments are synthetic.

^c^Natural DNA and RNA used in ligand screening experiments were pre-treated with nuclease (Benzonase, Merck).

Several chemotypes of LPS, including Rc, Rd, Re, Lipid A and FITC-LPS, and Pam3CSK4, a synthetic lipopeptide, were tested. Similar peak shifts in gel-filtration and dimer formation confirmed by EM indicated conformational change upon LPS’s other chemotypes binding (Supplementary Fig. 2A). The absorption peak of FITC at 490 nm (Supplementary Fig. 2C) of NLRP6 complexed with FITC-LPS suggests direct binding.

A 3D EM density map of the NLRP6 dimer was reconstructed using 5495 particles and showed a C2 symmetric shape (Fig. 2d,e and Supplementary Fig. 2D,E). The overall structure of the NLRP6 dimer is boat shaped and two apparent caves in both boat rails indicate the possible location of two LRR domains in the dimer. An initial attempt to fit our NLRP6 monomer structural model into the dimer density map failed, so we generated a model for the NLRP6 dimer by rotating the LRR domain around the NOD domain as suggested by the NLRC4 structure^23^, and this fit the 3D-EM density dimer map better (Fig. 2f and Supplementary Fig. 2F). In the homodimer model, two LRR domains seem interact with each other in an antiparallel manner, forming the major interface for dimerization. Two additional density patches appear at the head-tail interface of two LRR domains and might potentially form a LPS binding site (Supplementary Fig. 2G). The locations of these potential LPS binding sites are similar to that for the TLR4-MD-2 complex^26^.

### The NLRP6 homodimers assemble into higher oligomers by ATP stimulation

**Figure 3:**
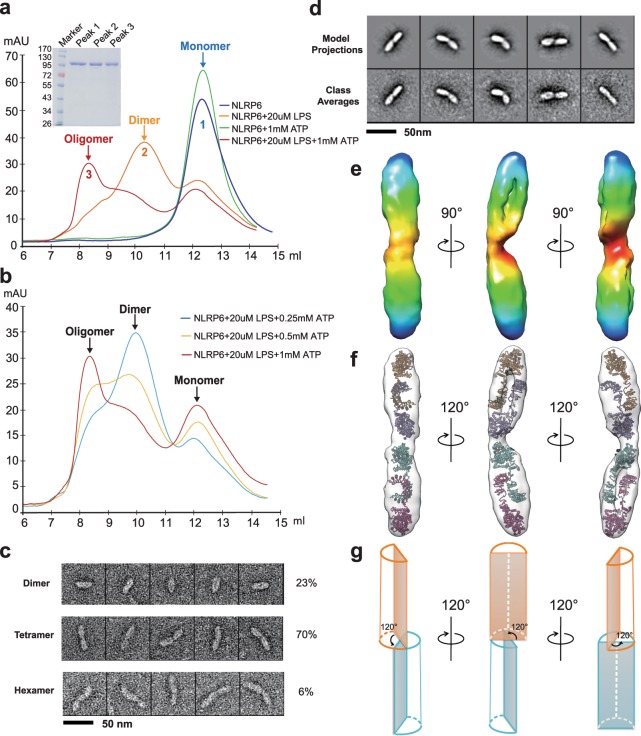
LPS-induced NLRP6 dimers assemble into higher molecular oligomers in the presence of ATP. (**a**) Elution profiles of NLRP6 monomer (blue), NLRP6 monomer incubated with ATP (green), NLRP6 dimer (incubated with LPS, orange) and NLRP6 oligomer (incubated with both LPS and ATP, red) in Superdex 200 size exclusion chromatography. Panel insert shows the SDS-PAGE of protein fractions in peak 1, 2 and 3. (**b**) The formation of NLRP6 oligomers is ATP concentration dependent. (**c**) Representative particles of various NLRP6 oligomers in Peak 3. (**d**) Comparison of representative experimentally determined class averages (below) to 2D reprojections of the reconstructed 3D volume (above) of NLRP6 tetramer. (**e**) 3D reconstruction of NLRP6 tetramer, as shown in three orientations. Two boat shaped dimer units link together in a head-to-head manner. (**f**) Model fitting of NLRP6 tetramer with the computational model of NLRP6 dimer. (**g**) A cartoon model showing a 120° anti-clock deflection between two dimer units. See also Supplementary Fig. 3, Supplementary Movie 1.

NLRs belong to the signal transduction ATPase with numerous domains (STAND) subfamily and NLR activation may be accompanied by the exchange of ADP for ATP through the NOD domain^24^. The roles of nucleotide binding in NLR activation were addressed with NLRP6. No detectable change appeared in the gel-filtration profile when the NLRP6 monomer was treated with ATP (Fig. 3a). However, in the presence of both ATP and LPS, NLRP6 showed additional peak shifts suggestive of oligomerization (Peak 3, Fig. 3a). Formation of the NLRP6 oligomer is ATP concentration-dependent and the oligomer ratio is positively correlated with ATP concentration (Fig. 3b). NLRP6 dimer formation following LPS binding and ATP-induced oligomerization are consistent with the two-step activation mechanism suggested for NLRP1 and NLRP3^5,27^. However, the asymmetric peak of NLRP6, presumed to arise from oligomer heterogeneity, suggests a more dedicated activation mechanism. Preliminary EM analysis of NLRP6 preparation with 1 mM ATP (Peak 3, Fig. 3a and Supplementary Fig. 3A) appears to be a mixture of NLRP6 oligomers shaped like ‘linked-sausages’ of various lengths. The number of distinguishable shrinked links observed in raw images enabled us to classify all the oligomers as dimer (23%), tetramer (70%), hexamer (6%), octamer (<1%) and even higher oligomers (<1%) (Fig. 3c). The transition of NLRP6 from dimeric to oligomeric state is driven by non-specific nucleotide binding as demonstrated by ATP, GTP, CTP, TTP, and deoxy-ATP (dATP) treatment (Supplementary Fig. 3D), which is in agreement with previous studies on the NOD domain.

### 3D-EM reconstruction of NLRP6 tetramer

Given that conformational heterogeneity of NLRP6 oligomerization may play an important role in NLR activation, we examined the structure of different fractions of NLRP6 oligomers. We reconstructed a 3D EM density map of the NLRP6 tetramer with 8783 particles and the reconstructed 3D volume fit experimentally-determined class averages (Fig. 3d and Supplementary Fig. 3B,C). The overall structure of the tetramer resembles a ‘∞’ whereby two boat-shaped dimer units are linked together in a head-to-head manner with 120° deflection in the central linkage (Fig. 3e–g and Supplementary Movie 1). The NLRP6 dimer structural model fit our tetramer density map, suggesting rigid body interaction of dimers (Fig. 3f and Supplementary Fig. 3E). Given that NLRP6 polymerization was induced by ATP binding via a potential nucleotides binding site located in the NOD domain, it is more likely that NOD domains are involved directly in dimer-dimer interaction. Adding a MBP tag to the N terminal of NLRP6 did not affect dimerization and further polymerization induced by LPS and ATP, which strongly indicates that the linear packing of NLRP6 leaves space for recruiting adaptor proteins through the PYD domain.

### 3D-EM reconstruction of NLRP6 hexamer

**Figure 4:**
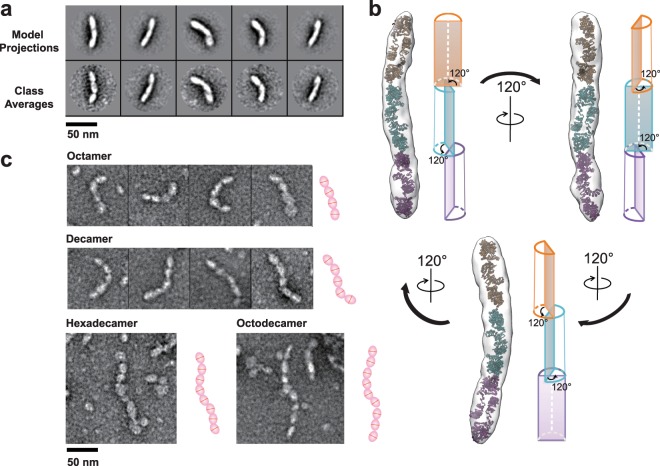
Higher molecular weight NLRP6 oligomers. (**a**) Comparison of representative experimentally determined class averages (below) to 2D reprojections of the 3D-reconstructed hexamer volume (above). (**b**) Model fitting of NLRP6 hexamer with the NLRP6 dimer model and a cartoon showing a 120° anti-clock deflection. (**c**) Representative particles of NLRP6 octamer, decamer, hexadecamer and octadecamer. An obvious shrinked link exists between each two neighboring dimer units. See also Supplementary Fig. 4, Supplementary Movie 2.

The overall shape of the hexamer (Fig. 4a and Supplementary Fig. 4) resembles three dimers linked together in a line, suggesting the dimer is also the building block of the hexamer through homo-oligomeric assembly. With the computational model of the NLRP6 dimer fitting the hexamer 3D EM density map, we obtained a NLRP6 hexamer structural model (Fig. 4b) and identified a shrinked link and a 120° anti-clock rotation between each two-neighboring dimer units (Fig. 4b and Supplementary Movie 2). From a structural viewpoint, the hexamer with 120° rotation between each dimer unit may represent a structural repeat unit in the assembly process.

The oligomerization of NLRP6 shows ATP concentration dependence (Fig. 3b). Particle numbers for the NLRP6 higher oligomers such as octamer and decamer were relatively limited (Fig. 4c), but significantly increased with higher ATP concentration. Hexadecamer and octadecamer were also observed under EM when ATP concentration increased to 2 mM (Fig. 4c), and their linked-sausage shape and the size of each unit suggest they assemble in a linear pattern using the dimer as building blocks.

### LPS induces co-localization of NLRP6 and ASC in cells

**Figure 5:**
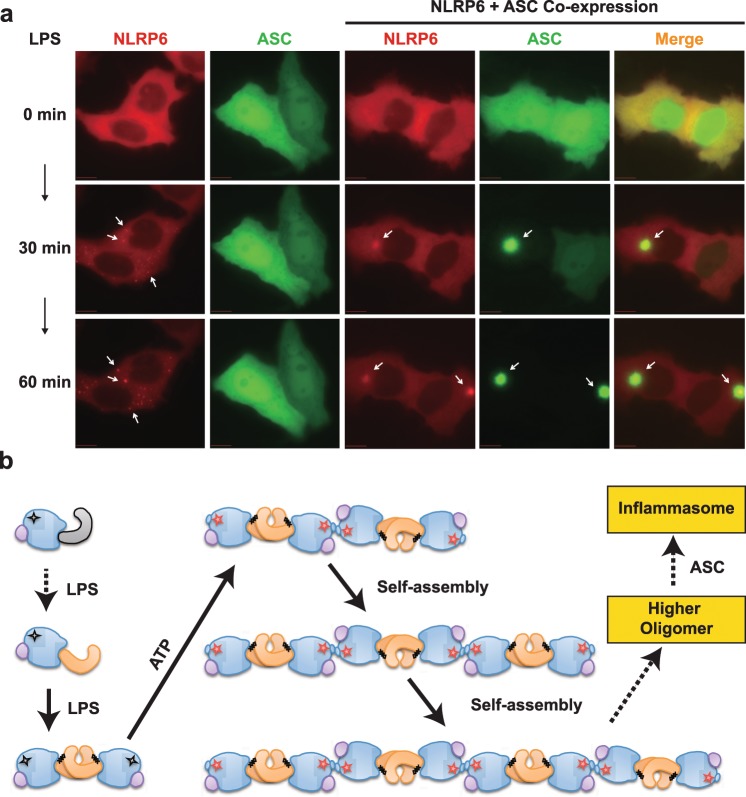
Co-localization of NLRP6 and ASC after LPS infection. (**a**) Live-imaging of fluorescent fused NLRP6-mCherry (red), ASC-GFP (green) and the merge in HeLa cells transfected with LPS for the indicated hours. Scale bars represent 10 um. (**b**) Possible step-by-step activation mechanism of NLRP6 based on oligomerization. LPS is able to bind NLRP6 directly and induces its conformational change and dimerization. In the presence of ATP, the NLRP6 homodimer further self-assembles into tetramer, hexamer and even larger oligomers, providing a novel linear molecular platform for the recruitment of signaling partners such as ASC, and may then assemble into inflammasome or other signal complexes. See also Supplementary Figs. 5 and 6.

The DeltaVision live cell imaging system was applied to confirm the LPS binding of NLRP6 and elucidate NLRP6 oligomerization and ASC recruitment in cells (Fig. 5a). HeLa cells expressing either NLRP6 or ASC, or both, were treated with LPS. Prior to LPS treatment, both NLRP6 and ASC were evenly distributed in the cytosol; ASC was also found in the nucleus. Following LPS treatment, cells expressing NLRP6 showed dotted-like aggregation in the cytosol consistent with our biochemical and structural findings. Cells expressing ASC alone, NLRP6 without LRR domain (NLRP6△LRR) and NLRP3 showed no response to LPS (Supplementary Fig. 5). Interestingly, cells expressing both full-length NLRP6 and ASC showed dramatic aggregation and co-localization of NLRP6 and ASC upon LPS treatment, suggesting LPS specifically induces self-assembly of NLRP6 through LRR domain and the subsequent recruitment of ASC, leading to a higher oligomeric structure in cells. Coincidentally, studies on NLRP3, NLRC4, AIM2, and NLRP1 indicate similar ASC specks recruitment during inflammasome assembly^5,28–30^. Different chemotypes of LPS (Rc, Rd, Re LPS, lipid A and pam3csk4) showed similar induction of NLRP6 aggregation and subsequently ASC recruitment (Supplementary Fig. 6), consistent with our biochemical results.

## Discussion

LPS is a glycolipid located in the outer membrane of gram-negative bacteria. Extracellular LPS is thought to be detected exclusively by Toll-like receptor 4 (TLR4) and its co-receptors^26,31^, and recent studies propose that LPS can be sensed by inflammatory caspases in the cytoplasm of macrophages^32,33^. Our results provide biochemical and cellular evidences of an alternative intracellular LPS recognition mechanism through NLRP6. NLRP6 has been proposed as a multifaceted NLR, regulating both inflammasome-dependent and inflammasome-independent functions^12,13,19,22^. Recent studies found NLRP6 was involved in the regulation of autophagy and mucus granule exocytosis in goblet cells, orchestration of antimicrobial peptide secretion, epithelial regeneration, viral RNA recognition, and regulation of inflammatory signaling in myeloid cells. Currently the leading view is that each of these functions should occur in a different cell type^20,34^.

Our data confirm that LPS binds directly to the NLRP6 monomer and induces its dimerization, similar to TLR4. Considering NLRP6 is expressed highly in the intestinal epithelium and with low expression in microphages^13,17^, our results suggest that NLRP6 might act as a LPS sensor in the intestine where a high abundance of commensal bacterial exist, thus playing a crucial role in maintaining intestinal homeostasis. Cytosolic LPS may be recognized differently in macrophages^33^ and in intestine epithelium, consistent with different expression patterns for NLRP6. Mice lacking NLRP6 feature improved resistance to systemic infection with gram-negative Enterobacteriaceae while being more susceptible to intestinal infection with members of the same bacterial family emphasizes the concept that NLRP6 may perform highly differential functions in a cell type- and context specific manner^16,19,34^. Further, the oligomerization of NLRP6 triggered by LPS and ATP in a characteristic two-step mechanism appears to be a novel linear molecular platform for the recruitment of signaling partners such as ASC, and may then assemble into inflammasome or another filamentous signaling complex^35^.

Our activation model of NLRP6 is in contrast to suggested ring-like inflammasome arrangements based on the Apaf-1 apoptosome structure^36^, and also the recent filament model in which NLRP6 forms into filament with a PYD core surrounded by the NBD and the LRR domain in absence of ligand^37^. Indeed, due to a rigid and stable dimer formation upon high affinity ligand binding, a ring-like NLR arrangement consisting of seven re-organized monomers seems to be implausible for LPS-induced NLRP6 polymerization and activation, the process identified here appears to be a precise step-by-step mechanism (Fig. 5b). The ratio of various oligomers was influenced by the concentration of ATP, but it remains unclear if a threshold value for NLRP6 oligomer size (or length) exists and if reaching this threshold triggers downstream signaling, such as autophagy for degradation^35^ (Fig. 5b). The aggregation of NLRP6 and the co-localization of NLRP6 and ASC upon LPS observed in cells clearly show that oligomerization of NLRP6 occurs, recruitment of ASC is initiated, and this may lead to the assembly of inflammasomes or other types of signal complexes (Fig. 5).

Given historical difficulties in identifying ligands and activation pathways for NLRs using animal models, we used a unique biochemical approach to identify direct interaction between NLRs and selected pathogen-associated molecular patterns (PAMPs), followed-up by live cell imaging. Difficulties associated with NLR expression and purification have limited the past application of this approach because it requires monomeric (ligand unbound status) full-length NLR protein to detect direct ligand binding and conformational changes and large quantities of purified NLR proteins to perform multiple experiments. However, we successfully obtained homogenous NLRP6 monomer at 2 mg/L with 99% purity. Our methods were successful in identifying NLR binders/ligands with certainty, and should be viewed as a valuable supplement to traditional ligand screening.

## Methods

### Expression and purification of full-length NLRP6 monomer

Full-length NLRP6 fused with N-terminal His-tag followed by a maltose binding protein (MBP) tag were expressed in E. coli, and purified by Ni-NTA (QIAGEN) affinity chromatography followed by Amylose affinity chromatography (NEB). After HRV3C digestion, high purity NLRP6 protein preparations in the putative ligand binding oligomeric state were obtained. Using protein solubility test^38^ and analytical gel filtration, an appropriate buffer condition (20 mM MES pH 6.5, 0.5 M NaCl, 5% glycerol and with 0.2‒0.5 M Arginine) was identified out to keep NLRP6 in a monomeric state.

### Screening of NLRP6 potential ligands

To identify the potential ligand of NLRP6, a panel of microbial components including LPS(Ra)^26^ were selected to be incubated with NLRP6 monomer in 20:1 molar ratio, and then loading to a Superdex 200 10/300 column (GE Healthcare).

### Electron microscopy and image processing

Preparation of negatively stained samples and image acquisition were as described elsewhere^39^. The image processing was using EMAN and EMAN2.

### SPR experiment

In order to investigate the interaction between LPS and NLRP6, a kinetics assay was performed at 298 K using a Surface Plasmon Resonance (SPR) Biacore 3000 machine (GE Healthcare, Uppsala, Sweden). Kinetic profiling was performed using the single cycle kinetics method^25^. Data were analyzed using Biacore 3000 and fit to a 1:1 Langmuirbinding model.

### Live cell imaging

Live cell imaging was performed using a DeltaVision live cell imaging system (Applied Precision) equipped with an Olympus IX-71 inverted microscope and a 100×, 1.40 N.A. oil objective. Images were captured with 100 ms exposure times in 30 min intervals by a CoolSnap HQ2 CCD camera, and different Z sections were projected by SoftWorx suite.

## Supplementary information

Supplementary Information   

Supplementary Information 1 

Supplementary Information2

## Data Availability

We declare that all the data supporting the findings of this study are available within the paper and the Supplementary Information files.
