# Supplementary material for: NLRP6 self-assembles into a linear molecular platform following LPS binding and ATP stimulation
Source: Sci Rep. 2020 Jan 13;10:198. doi: 10.1038/s41598-019-57043-0 (PMC6957519; doi:10.1038/s41598-019-57043-0)
Supplement: Supplementary file 3 — Supplementary Information2 [file 41598_2019_57043_MOESM3_ESM.pdf]

NLRP6 self-assembles into a linear molecular platform following LPS binding and ATP stimulation

Fangwei Leng[1,2,3,8], Hang Yin[2,4,8], Siying Qin[2], Kai Zhang[1], Yukun Guan[5], Run Fang[5], Honglei Wang[6], Guohui Li[6], Zhengfan Jiang[5], Fei Sun[1], Da-Cheng Wang[1]* & Can Xie[2,7]*

[1]National Laboratory of Macromolecules, Institute of Biophysics, Chinese Academy of Sciences, Beijing, 100101, China

[2]State Key Laboratory of Membrane Biology, Laboratory of Molecular Biophysics, School of Life Sciences, Peking University, Beijing 100871, China

[3]Department of Biological Chemistry and Molecular Pharmacology, Harvard Medical School, Boston, MA 02115, USA;

[4]Department of Medicinal Chemistry and Molecular Pharmacology, Purdue University, West Lafayette, IN, 47907, USA

[5]State Key Laboratory of Protein and Plant Gene Research, Peking-Tsinghua Center for Life Sciences, College of Life Sciences, Peking University, Beijing 100871, China

[6]Laboratory of Molecular Modeling and Design, State Key Lab of Molecular Reaction Dynamics, Dalian Institute of Chemical Physics, Chinese Academy of Sciences, Liaoning, 116023, China

[7]High Magnetic Field Laboratory, Hefei Institutes of Physical Science, Chinese Academy of Sciences, Hefei 230031, China

[8]Co-first author

*Correspondence: canxie@hmfl.ac.cn (C.X.), dcwang@ibp.ac.cn (D.C.W.)

Supplementary Movies

Supplementary Movie S1. Molecular Architecture of the NLRP6 tetramer, related to Figure 3.

This movie is related to Figure 3. It shows the reconstructed 3D EM map of the NLRP6 tetramer and model fitting with the NLRP6 dimer model. There is a 120 °anti-clock deflection between two dimer units.

Supplementary Movie S2. Molecular Architecture of the NLRP6 hexamer, related to Figure 4.

This movie is related to Figure 4. It shows the reconstructed 3D EM map of the NLRP6 hexamer and model fitting with the NLRP6 dimer model. There is a 120 °anti-clock deflection between each two neighboring dimer units, analogous to the NLRP6 tetramer.

Supplementary Figures

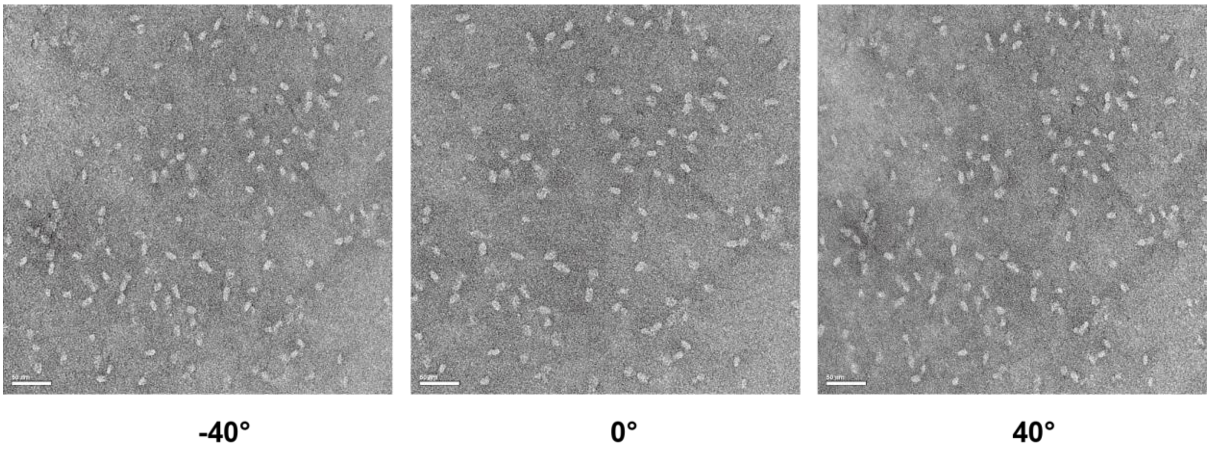

Supplementary Figure S1. EM micrographs of NLRP6 monomer, related to Figure 1.

Representative negative-staining EM raw images of NLRP6 monomer (50000x. Bars: 50 nm.

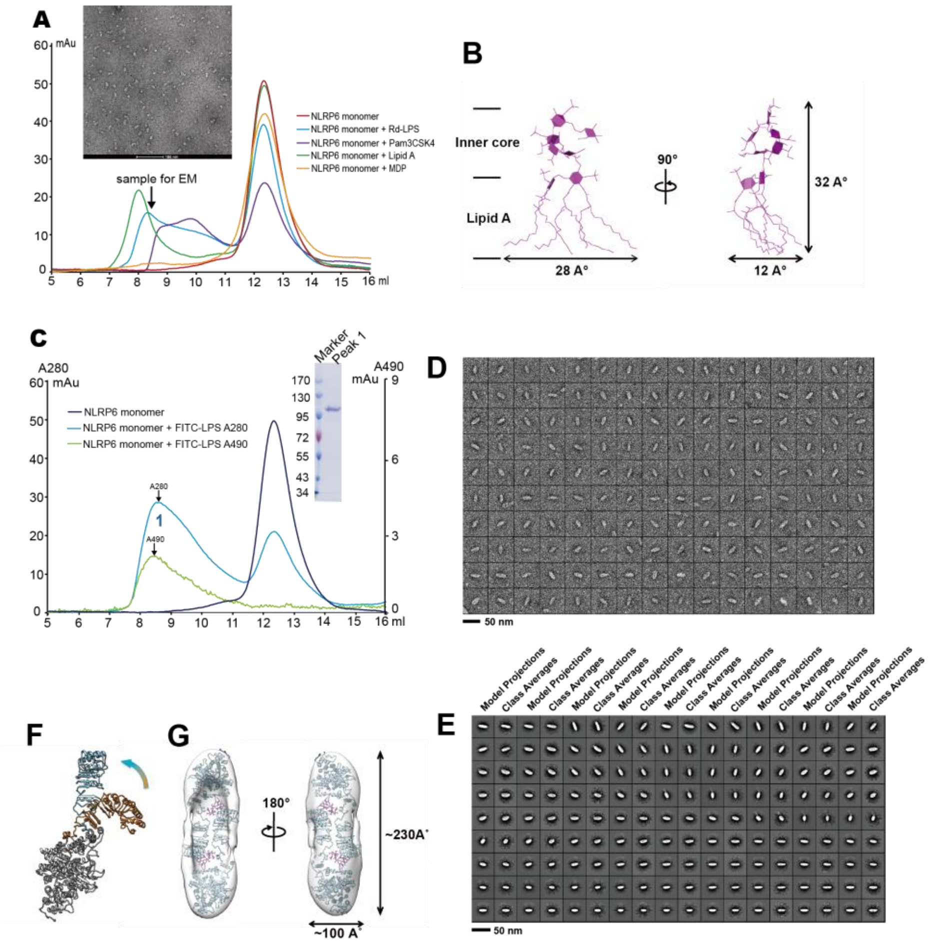

Supplementary Figure S2. EM micrographs of NLRP6 dimer, related to Figure 2.

(A) Gel filtration analysis of NLRP6 monomer (red), and NLRP6 monomer incubated with LPS Rd (blue), pam3CSK4 (purple), Lipid A(green) and MDP (orange) in Superdex 200 size exclusion chromatography. Panel insert shows the representive raw image of other chemotypes of LPS-induced NLRP6 dimer. (**B**) structure of LPS (Ra), based on the crystal structure of the TLR4/MD-2/Ra LPS complex (PDB ID 3FXI). **(C)** The FITC absorption peak at 490 nm reveal a direct interaction between NLRP6 and FITC-LPS. (D) Presentative raw particles of the NLRP6 dimer. **(E)** comparison of representative experimentally-determined class averages to 2D reprojections of the reconstructed 3D volume of dimer. Bars: 50 nm. **(F)** The putative conformational change upon ligand (LPS) binding. The final NLRP6 dimer model was aligned to the previously fitted monomer model based on NOD and PYD domains. The different orientation of LRR domains is highlighted in cyan (monomer) and orange (dimer). **(G)** Model fitting of NLRP6 dimer. The final NLRP6 dimer model was fit into the 3D EM density map, and suggested putative LPS binding sites are shown near the head-tail interface of two LRR domains in the NLRP6 dimer.

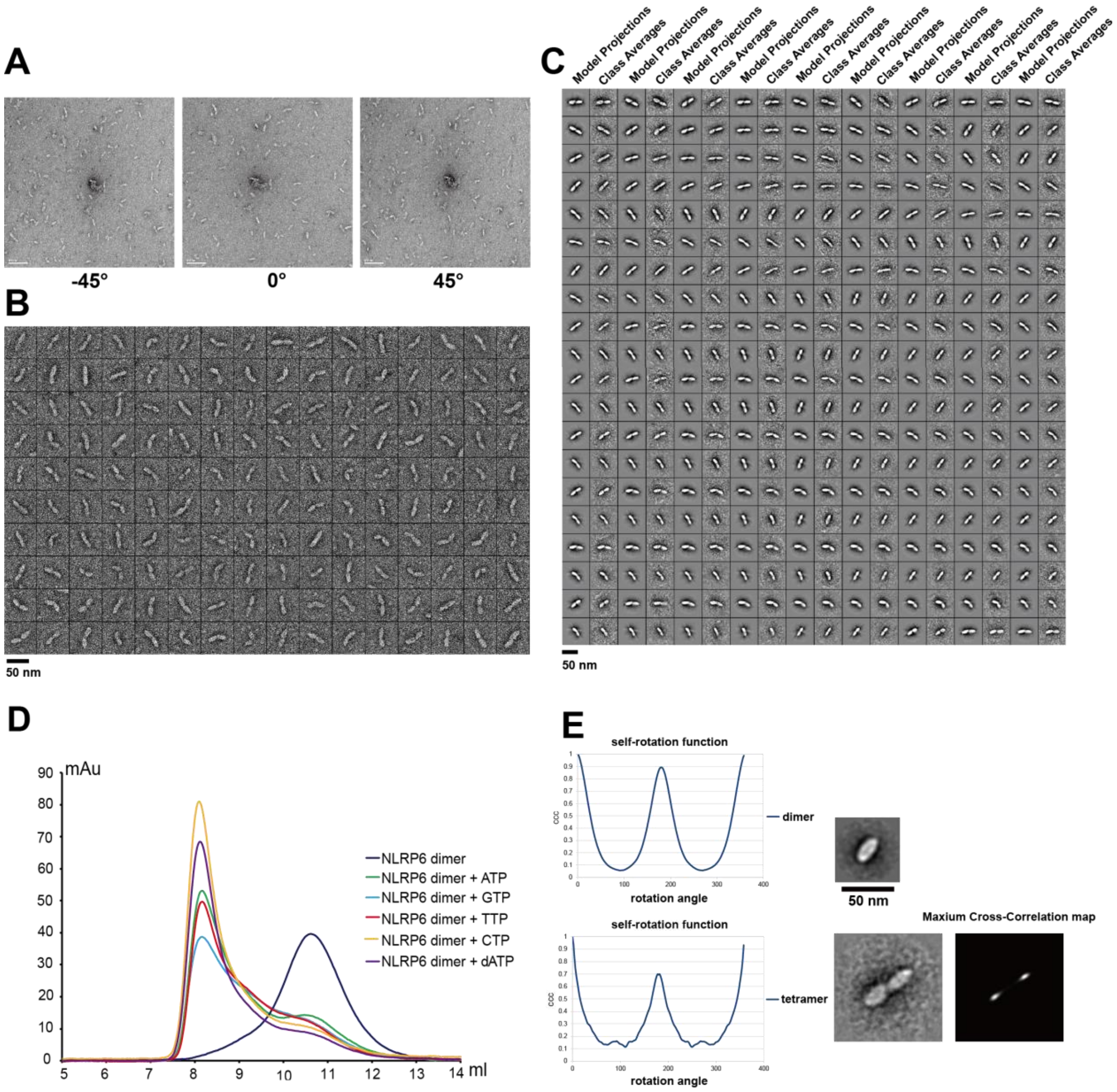

Supplementary Figure S3. EM micrographs of NLRP6 oligomers, related to Figure 3. **(A)** Representative negative-staining EM raw images of NLRP6 oligomers (29000x). Bars: 100 nm. The major population of NLRP6 oligomes induced by both LPS and ATP are mainly dimers, tetramers and hexamers, as shown in these raw images. **(B)** Representative raw particles of the NLRP6 tetramer. An obvious shrinked link exist in the center and between two dimer units. **(C)** Comparison of representative experimentally-determined class averagesto 2D reprojections of the tetramer 3D volume. **(D)** The oligomerization of NLRP6 dimer induced by NTPs and dATP. **(E)** Self-rotation function of NLRP6 dimer and tetramer, and the maxium cross-correlation map.

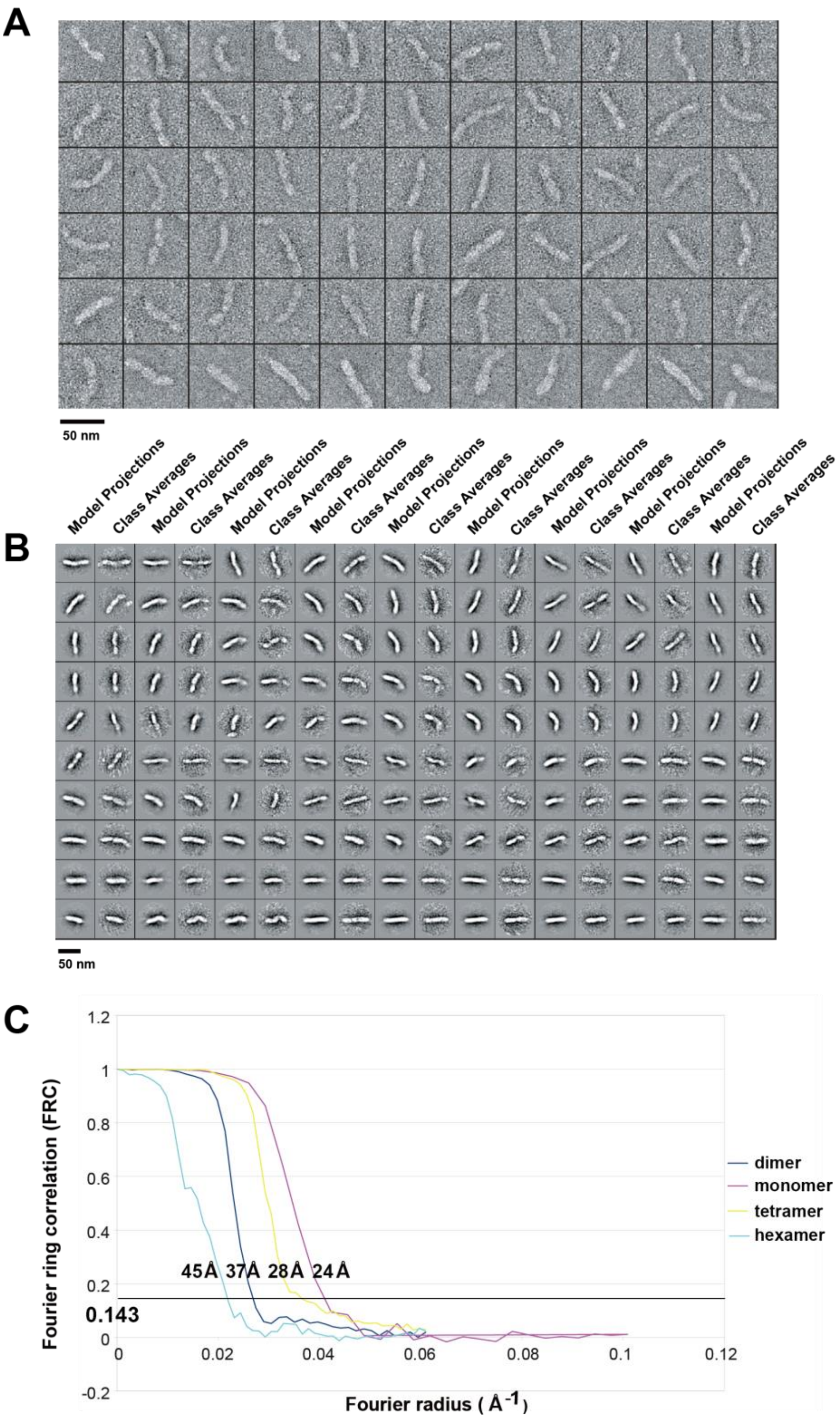

Supplementary Figure S4. EM micrographs of NLRP6 hexamer, related to Figure 4. **(A)** Representative raw particles of the NLRP6 hexamer showing a linked-sausage shape. **(B)** Comparison of representative experimentally-determined class averages to 2D reprojections of the reconstructed 3D volume of hexamer. **(C)** Resolution of various 3D-EM reconstructions.

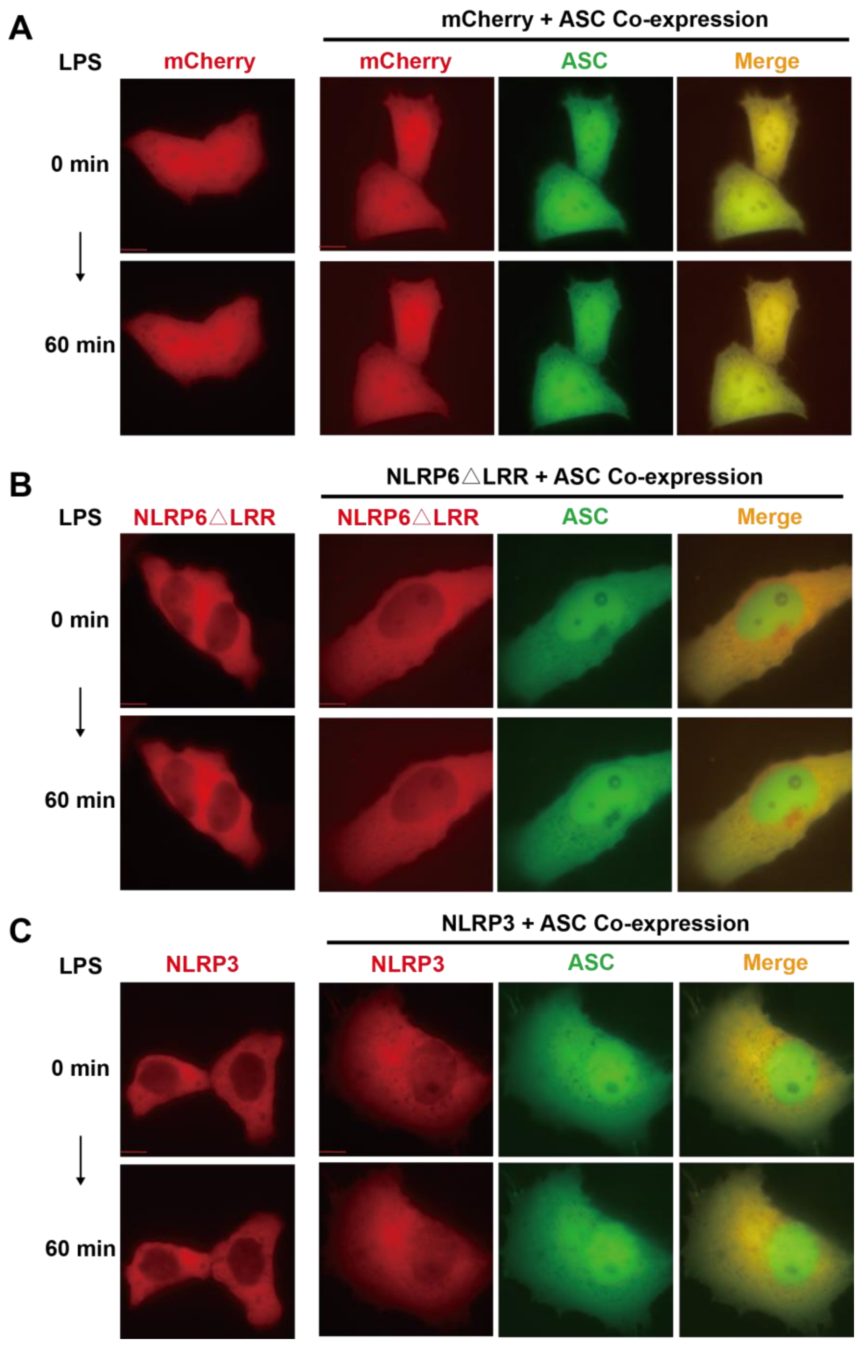

Supplementary Figure S5. mCheery, NLRP6 without LRR domain and NLRP3 has no response to LPS stimulation in live cells, related to Figure 5.

(A) Live-imaging of mCherry (red), ASC-GFP (green) and the merge in HeLa cells transfected with LPS for the indicated hours. Scale bars = 10 um. **(B)** Live-imaging of NLRP6 (PYD+NOD)-mCherry (red), ASC-GFP (green) and the merge in HeLa cells transfected with LPS for the indicated hours. Scale bars = 10 um. **(C)** Live-imaging of NLRP3-mCherry (red), ASC-GFP (green) and the merge in HeLa cells transfected with LPS for the indicated hours. Scale bars = 10 um.

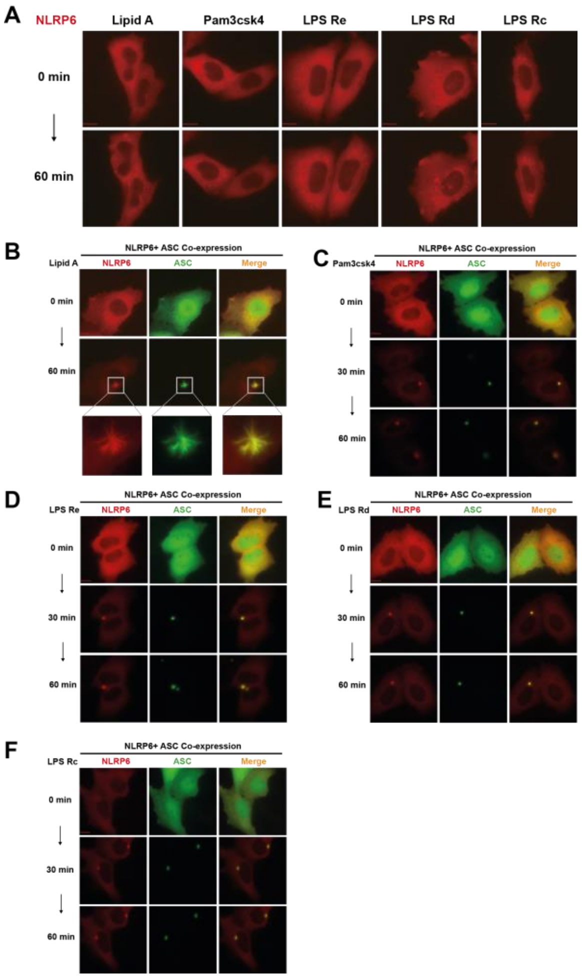

Supplementary Figure S6. Other chemicotypes of LPS are capable of inducing the dotted-like aggregation of NLRP6 and subsequently recruitment of ASC in the cytosol, related to Figure 5.

(A) Live-imaging of NLRP6-mCherry (red) in HeLa cells transfected with Lipid A, pam3csk4, LPS Re, LPS Rd, LPS Rc respectively for the indicated hours. Scale bars = 10 um. **(B)** Live-imaging of fluorescent fused NLRP6-mCherry (red), ASC-GFP (green) and the merge in HeLa cells transfected with lipid A for the indicated hours. Scale bars = 10 um. **(C)** Live-imaging of NLRP6-mCherry (red), ASC-GFP (green) and the merge in HeLa cells transfected with pam3csk4 for the indicated hours. Scale bars = 10 um. **(D)** Live-imaging of NLRP6-mCherry (red), ASC-GFP (green) and the merge in HeLa cells transfected with LPS Re for the indicated hours. Scale bars = 10 um. **(E)** Live-imaging of NLRP6-mCherry (red), ASC-GFP (green) and the merge in HeLa cells transfected with LPS Rd for the indicated hours. Scale bars = 10 um. **(F)** Live-imaging of NLRP6-mCherry (red), ASC-GFP (green) and the merge in HeLa cells transfected with LPS Rc for the indicated hours. Scale bars = 10 um.

Supplementary Tables

Supplementary table S1. Negative staining EM reconstructions and data entries, related to Figure 1.

Sample name	MAG#	Binning	Box size	Pixel size	Number of particles used
monomer	50000	2	128	4.80	16128
dimer	29000	2	160	8.28	5495
tetramer	25000	2	200	9.6	8783
hexamer	25000	2	200	9.6	2873

#MAG, nominal magnification.

Supplementary table S2. Kinetics and affinity constants for LPS binding to NLRP6, related to Figure 2.

Association rate $k_a(M^{-1}s^{-1})$	Dissociation rate k_d $(M^{-1}s^{-1})$	Binding affinity K_d (M)
1.47×10^{2}	1.11×10^{-5}	7.53×10^{-8}

Extended experimental procedures

Expression and purification of full-length NLRP6 monomer

To obtain full-length NLRs protein preparation for ligand screening, all 22 full-length human NLRs fused with N-terminal His-tag followed by a maltose binding protein (MBP) tag were cloned into our customized expression vectors, driven by a cold-shock promoter (*cspA*) and a *lac* operator, and expressed in *Escherichia coli* strain BL21(DE3). A HRV3C recognition sequence was inserted between the affinity tag and the NLRs. After 20 h of incubation at 288 K cells were harvested, resuspended and sonicated in the lysis buffer (20 mM Tris pH 8.0, 150 mMNaCl, 10 mM imidazole, 10 mM 2-mercaptoethanol) with complete protease inhibitor cocktail (Roche). Insoluble debris was removed by centrifugation for 30 min at 16000 g and 277 K. Recombinant proteins were purified from supernatant by Ni-NTA (QIAGEN) affinity chromatography and Amylose affinity chromatography (NEB). After HRV3C digestion to remove N-terminal His tag and MBP tag, high purity NLRP6, NLRP10, NLRX1 protein preparations were obtained. However, all these preparations eluted as oligomer in size exclusion chromatography, indicating a possible activation by binding

components from bacterial (Fig. 1b). To obtain ligand free and inactive monomeric NLRs, different purification strategies and buffer conditions have been tested for each NLRs. One condition (0.2–0.5M Arginine) identified by protein solubility test [1] and analytical gel filtration worked for NLRP6. Under this condition, the NLRP6 monomer can be released from previous oligomers and remain in monomeric state (Fig. 1c). The final full-length NLRP6 protein (1-892) preparation applying to following experiments was 99% pure and eluted as homogeneous monomer from Superdex 200 10/300 column (GE Healthcare) in 20mM MES pH6.5, 0.5 M NaCl, 0.4 M Arginine and 5% glycerol.

Screening of NLRP6 potential ligands and LPS binding experiments

To identify the potential ligand of NLRP6, a panel of microbial components were selected to be incubated with NLRP6 monomer, including NOD1/NOD2 agonists [2], Ra/Rc forms of LPS [3], RNA [4], and both natural and synthetic DNA sequences [5] (Table 1). Prior to incubation, the ligand candidates were pre-treated as follows: (1) LPS was sonicated on ice at 200 W using 4 s pulses with 8 s intervals for 10 min; (2) DNA and RNA extracted from *E. coli* were treated with nuclease (Benzonase, Merck) so as to degrade them into small size; (3) for the rest of other ligand candidates, they were dissolved in the indicated amounts of H_2O. For each ligand screen test, 0.1 mg NLRP6 was incubated with ligand candidates as shown in table S1 in 1:20 molar ratio at 25 ℃ for 3 h, and then loading to a Superdex 200 10/300 column (GE Healthcare). The elution volume of NLRP6 peak in each experiment was compared with the peak of untreated NLRP6 monomer as a reference. Each experiment was repeated three times.

After LPS was identified as a potential ligand for NLRP6, different chemotypes of LPS, including Ra, Rc, Rd, Re, Lipid A, FITC-LPS, and Pam3CSK4, a synthetic lipopeptide, were tested and compated in ligand binding experiments. Among all these LPS chemotypes, Ra and Re LPS designate the mutants with the longest and shortest polysaccharide chain lengths, respectively. After sonication, Ra LPS solution become transparent however Re LPS remained turbid, suggesting that Ra LPS form small and homogeneous micelles, and Re LPS form larger and highly heterogeneous aggregates owing to their greater hydrophobicity versus hydrophilicity ratio. Lipid A appeared to be the most turbid solution after sonication, consistent with the idea that the self aggregation of LPS is generally caused by the lipid A component of the molecule. The self-aggregation different LPS chemotypes may contribute partially to the peak shift in analytical gel filtration (Supplementary Fig. 2A-C). However, the EM image and measurement of all the LPS chemotypes treated NLRP6 “aggregates” showed homogeneous dimer formation, confirmed oligomerization of NLRP6 did not occur

(Supplementary Fig. 2A), which is in contrast with the ATP and LPS induced peak shift. Referring to previous work, Ra LPS forms small and homogeneous micelles and was successfully used in crystallization of TLR4 [3]. Thus we chose the Ra form of for the electron microscopic experiments and SPR experiments.

Electron microscopy and image processing

NLRP6 monomer, dimer (incubated with LPS) and oligomer (incubated with LPS plus ATP) was subjected to Superdex 200 gel filtration chromatography (GE Healthcare). The NLRP6 protein fractions (Fig. 3a) were immediately diluted to 5–10 μg/ml and applied to grids after elution. Preparation of negatively stained samples and image acquisition were as described elsewhere [6]. About 100 particles of both monomers and tetramers were manually picked first. These particles were then classified and averaged to generate five templates for automatic particle picking, using the GPU-accelerated program GautoMatch (http://feilab.ibp.ac.cn/LBEMSB/AutoMatch.html). Raw particles of dimers, tetramers and hexamers were picked individually (Table S1). The defocus value of each micrograph was determined by CTFFIND3 [7] and the CTF was corrected by applying phase flipping to each micrograph using applyctf in EMAN [8]. The reference-free two-dimensional classification was performed using e2refine2d.py in EMAN2 [9]. Initial models for monomers, dimers, tetramers and hexamers were built using random conical tilt method in EMAN2 [9]. Before model refinement, multi-reference refinements (e2refinemulti.py in EMAN2) were taken for the data sets for monomers and tetramers to coarsely classify them into sub-classes. Only the best classes of monomers and tetramers were kept in order to reduce heterogeneity during further refinement. Since there were fewer particles for dimers and hexamers this procedure was not carried out on these data sets. Model refinements were then performed for all the data sets separately using e2refine.py. All models were refined without any symmetric restraint at first. Analysis of the symmetry-free models by self-rotation suggested that dimer and tetramers adopted good C2 symmetry. Therefore, both the dimer and tetramer were refined under restraint of C2 symmetry. All models were validated by projection matches and tilt-pair parameter plots. All parameters for image processing, including imaging magnification, box sizes, binning sizes, pixel or voxel sizes, particle numbers and final resolutions are listed in Table S1.

SPR experiment

In order to investigate the interaction between LPS and NLRP6, a kinetics assay was performed at 298 K using a Surface Plasmon Resonance (SPR) Biacore3000 machine (GE Healthcare, Uppsala, Sweden). A running buffer containing 50 mM MES pH 6.5, 500 mM

NaCl, 0.005% tween-20 was prepared, vacuum filtered and degassed immediately prior to the experiment. NLRP6 monomer from gel filtration chromatography was dissolved in 10 mM sodium acetate pH 4.5 with a concentration of 5 μg/ml and immobilized on a CM5 sensor chip with 1300 RU. The Ra chemotype of *E. coli* LPS (Sigma, L9641) was dissolved in running buffer at a concentration of 1 mg/ml, after sonicated on ice so as to separate LPS into small and homogeneous micelles.

Kinetic profiling was performed using the single cycle kinetics method [10]. In each analysis cycle, increased LPS concentrations were injected consecutively over the NLRP6 surfaces and a reference blank flow cell at a flow rate of 10 μl/min. LPS was diluted in the running buffer to four different concentrations in the range 0.9 μM to 25 μM and injected for 60 s at each concentration. After the last injection, the running buffer was run alone for 10 min. Data were analyzed using Biacore 3000 and fit to a 1:1 Langmuirbinding model.

Live Cell Imaging

Full length human NLRP6, NLRP3, mCherry, NLRP6 with LRR domain deleted (NLRP6△LRR) and ASC gene were cloned into expression vector pmCherry-N1 and pEGFP-N1 (Clontech) respectively. For real-time recording of live cell images, HeLa cells were transfected with fluorescent conjugated NLRP6 (NLRP6-mCherry), NLRP3-mCherry, mCherry, NLRP6△LRR-mCherry and/or ASC (ASC-GFP) for 18 hours. Then the transfected cells were placed onto a heated sample stage within a heated chamber (37 ℃). Following LPS (including different chemotypes of LPS) transfected with Lipofectamine 2000 (invitrogen), live cell imaging was performed using a DeltaVision live cell imaging system (Applied Precision) equipped with an Olympus IX-71 inverted microscope and a 100×, 1.40 N.A. oil objective. Images were captured with 100 ms exposure times in 30 min intervals by a CoolSnap HQ2 CCD camera, and different Z sections were projected by SoftWorx suite.

Supplementary References

1 Collins, B. K., Tomanicek, S. J., Lyamicheva, N., Kaiser, M. W. & Mueser, T. C. A preliminary solubility screen used to improve crystallization trials: crystallization and preliminary X-ray structure determination of Aeropyrum pernix flap endonuclease-1. *Acta crystallographica. Section D, Biological crystallography* **60**, 1674-1678, doi:10.1107/S090744490401844X (2004).

2 Kufer, T. A., Banks, D. J. & Philpott, D. J. Innate immune sensing of microbes by Nod proteins. *Annals of the New York Academy of Sciences* **1072**, 19-27, doi:10.1196/annals.1326.020 (2006).

3 Park, B. S. *et al.* The structural basis of lipopolysaccharide recognition by the TLR4-MD-2 complex. *Nature* **458**, 1191-1195, doi:10.1038/nature07830 (2009).

4 Hong, M., Yoon, S. I. & Wilson, I. A. Structure and functional characterization of the RNA-binding element of the NLRX1 innate immune modulator. *Immunity* **36**, 337-347, doi:10.1016/j.immuni.2011.12.018 (2012).

5 Kawai, T. & Akira, S. The roles of TLRs, RLRs and NLRs in pathogen recognition. *International immunology* **21**, 317-337, doi:10.1093/intimm/dxp017 (2009).

6 Nishida, N. *et al.* Activation of leukocyte beta2 integrins by conversion from bent to extended conformations. *Immunity* **25**, 583-594, doi:10.1016/j.immuni.2006.07.016 (2006).

7 Mindell, J. A. & Grigorieff, N. Accurate determination of local defocus and specimen tilt in electron microscopy. *J Struct Biol* **142**, 334-347 (2003).

8 Ludtke, S. J., Baldwin, P. R. & Chiu, W. EMAN: semiautomated software for high-resolution single-particle reconstructions. *J Struct Biol* **128**, 82-97, doi:10.1006/jsbi.1999.4174 (1999).

9 Tang, G. *et al.* EMAN2: an extensible image processing suite for electron microscopy. *J Struct Biol* **157**, 38-46, doi:10.1016/j.jsb.2006.05.009 (2007).

10 Tang, Y., Mernaugh, R. & Zeng, X. Nonregeneration protocol for surface plasmon resonance: study of high-affinity interaction with high-density biosensors. *Analytical chemistry* **78**, 1841-1848, doi:10.1021/ac051868g (2006).
